# Antimicrobial resistance and ESBL profile of Uropathogens among pregnant women at Edna Adan Hospital, Hargeisa, Somaliland

**DOI:** 10.4314/ejhs.v31i3.22

**Published:** 2021-05

**Authors:** Hussein Mohamoud, Senait Tadesse, Awoke Derbie

**Affiliations:** 1 Department of Microbiology, Edna Adan University, Hargeisa, Somaliland; 2 Department of Medical Microbiology, College of Medicine & Health Sciences, Bahir Dar University, Ethiopia; 3 Center for Drug Development and therapeutics trial for Africa (CDTAfrica), Addis Ababa University, Ethiopia; 4 Department of Health Biotechnology, Biotechnology Research Institute, Bahir Dar University, Ethiopia

**Keywords:** Bacteriuria, Urinary tract infection, pregnant women, Antimicrobial resistance, Edna Adan Hospital, Hargeisa

## Abstract

**Background:**

The emergence and spread of antimicrobial resistance (AMR) among uropathogens is increasing, especially in resource limited settings due to a number of reasons. The production of Extended Spectrum β-Lactamase (ESBL) by some strains of E. coli and methicillin resistant Staphylococcus species, limits the choice of antimicrobials in the treatment of urinary tract infection (UTI) globally. However, little is known about the type of uropathogenes and their current AMR profile among pregnant women in Hargeisa, Somaliland.

**Methods:**

Clean-catch mid-stream urine samples were collected and processed for bacteriological culture and antimicrobial sensitivity testing (AST). Ceftazidime (30µg) and Cefotaxime (30µg) disks were used for ESBL screening as per CLSI guideline and each ESBL screening positive isolate were phenotypically confirmed by a combination disk test.

**Results:**

Among 376 study participants, 79 (21.0%) had significant bacteriuria (SBU). Majority at 58(73.4%) of the isolates were Gram-negative. The most predominant isolate was E.coli, 36(45.6%) followed by K. pneumonea 16(20.3%) and S. aureus at 9(11.4 %). The proportion of ESBL producing isolates was 25(32.9%). Gram-negatives showed high level resistance to ampicillin, amoxicillin, cefotaxime, and cephalexin at 87%, 85%, 57%, and 52%, respectively. Previous history of UTI, monthly income, educational status and having dysuria were significantly associated with SBU (p<0.05).

**Conclusion:**

Relatively high prevalence of uropathogens and an increased level of drug resistance were documented. Therefore, continued surveillance on the type of uropathogens and their AMR pattern is needed to ensure appropriate recommendations for the rational empirical treatment of UTI and for policy input.

## Introduction

Urinary tract infection (UTI) is an infection of some part of the urinary system, which includes kidneys, ureters, bladder or urethra. It is one of the most common health problems during pregnancy next to anemia. Bacteria that reside in the bowl are the main cause most UTIs (1-3). According to studies reported in different settings, antimicrobial resistance (AMR) rates among common uropathogens including *Escherichia coli, Klebsiella spp., P. mirabilis, P. aeruginosa, Staphylococcus spp.* and *Enterococcus spp.* have been increasing, and their susceptibility varies with time and place (5–10). Data on local bacterial etiology and their susceptibility profile is worthy to trace any change in time. Thus, timely updated reference for empirical therapy of UTI can be made. Absence and delay in the detection of AMR and Extended Spectrum β-Lactamase (ESBL) producing uropathogenic bacteria is associated with prolonged hospital stay, increased morbidity and mortality in pregnancy (3, 4). Regular evaluation and study of the prevalence, etiologic agents, and predisposing factors of UTI during pregnancy is essential in order to reduce its effects during pregnancy for both the maternal and fetal health (5–7).

Pregnancy causes a number of changes in the body that increase the likelihood of UTIs. Hormonal and mechanical changes can promote urinary stasis and vesicoureteral reflux. These changes, along with an already short urethra and difficulty with hygiene due to a distended pregnant belly, help make UTIs the most common bacterial infections during pregnancy. Untreated bacteruria during pregnancy is associated with risks to both the fetus and the mother, including pyelonephritis and adverse obstetric outcomes such as prematurity, low-birth weight and higher fetal mortality rates. Pregnant women with UTI are more likely to deliver premature or low-birth-weight infants (1–2).

In Edna Adan Hospital (EAH), same as other similar settings in Somaliland, routine culture and antibiotic susceptibility testing are not performed as an essential part of antenatal care and the most common treatment practice is based on empirical therapy. This may leads to the overuse of antibiotics and spread of resistant uropathogenes. Data on local bacterial etiology and their susceptibility profile is worthy to trace any change in time. Thus, timely updated reference for empirical therapy of UTI can be made (5–10). However, there is no previously published research on the prevalence, type of the isolates and their respective AMR profile among pregnant women in the study area. Moreover, there is huge information gap on ESBL producing bacterial uropathogens in the country. With this background information, this study was conducted aimed at assessing the bacterial, antimicrobial resistance and ESBL profile of uropathogens among pregnant women attending antenatal care at EAH, Hargeisa, Somaliland.

## Materials and Methods

**Study setting, design and period:** A hospital based cross-sectional study was conducted from 1 Feb 2019 to 30 May 2019 in EAH, Hargeisa, Somaliland. The hospital is located in Maroodi Jeex Region, the capital city of Somaliland known as Hargeisa. The city had one referral Hospital, two general Hospitals, seven health care centers and five private Hospitals and other several private clinics during the time of data collection. The AEH is one of the largest maternity private hospitals in the city which provides health services for the community especially, maternal and child health services, for patients from all parts of Somaliland and other neighborhood regions such as Puntland and Southern Somalia. Edna Hospital was founded by the famous lady in the context of Somalia and the world as well.

**Population, sample size and sampling technique:** A total of 384 study participants were included in the study. The study population was pregnant women attending ANC at EAH during the study period. Convenient sampling technique was used to enroll consecutive pregnant women attending antenatal care in the hospital during the study period who fulfilled the inclusion criteria. Pregnant women with or without symptoms of urinary tract infection who were willing to participate in the study were included. Those who took antibiotics two weeks before the time of data collection period were excluded.

**Data collection procedures:** Structured questionnaire that has been translated into the local language was used to collect demographic characteristics of the study participants and related clinical data. Pregnant women were screened for UTI clinically by health practitioners in charge of attending them. In addition, the types of isolated bacterial uropathogens and the ESBL producing bacteria from urine culture with their respect antimicrobial susceptibility profile were determined as per standard bacteriological protocol.

Urine sample collection and handling: After appropriate instruction pregnant women were given pre-labeled leak proof, wide mouth, and sterile, screw-capped plastic container to collect 5–10mL mid-stream urine (MSU) specimen. Then all samples were immediately transported to the bacteriology department at EAH for culture and antimicrobial susceptibility testing (AST). Using calibrated wire loop samples were inoculated in to Cystine Lactose Electrolyte Deficient medium (CLED). Cultures were incubated overnight in aerobic condition at 37°C for 24 hours and colonies were counted to check the presence of significant growth. Colony counts yielding growth of ≥10^5^ CFU/ml of urine was regarded as significant bacteriuria (SBU) (11–12).

Colonies from CLED were then sub cultured into MacConkey and blood agar plates, then incubated at 37°C for 24 hours. Identification of bacterial species was done using colony characteristics, gram staining and panel of biochemical tests following the standard procedure. The gram negative bacteria were identified by indole, H_2_S production in KIA agar, citrate utilization, urease test, motility test, oxidase and carbohydrate utilization tests. Catalase and coagulase tests were also employed to identify gram-positive isolates (11, 12).

**Antimicrobial susceptibility testing (AST):** The Kary-Baur disc diffusion method was used for AST on Muller Hinton agar (MHA) (Oxoid, Ltd, England) as per the Clinical Laboratory Standards Institute guideline (13). Identical 3–5 pure colonies from overnight cultured specimen were suspended in 5ml sterile nutrient broth (Oxoid, Ltd, England) and mixed thoroughly to make the suspension homogenous. The inoculum turbidity was adjusted to 0.5 McFarland standards. Then, the bacterial suspensions were seeded on the surface of the MHA using a sterile cotton swab. The antimicrobial impregnated disks were placed on the media using sterile forceps and plates were incubated at 37°C for 24 hours and the zone of inhibition was measured and interpreted as sensitive, intermediate and resistant as per the CLSI protocol. The following disks were used for gram negative bacteria; Amoxicillin (AML, 25µg), Ceftriaxone (CRO, 30µg), Cefotaxime (CTX, 30µg), Amoxicillin-clavulanic acid (AMC, 20/10µg), Nitrofurantoin (F, 300µg), Norfloxacin (NOR, 10µg), Cephalexin (CN, 30µg) and Ceftazidime (CAZ, 30µg). Similarly, Ampicillin (AMP, 10µg), Norfloxacin (NOR, 10µg), Cefotaxime (CTX, 30µg) and Amoxicillin (AML, 25µg), were also used for gram-positive isolates. These disks were selected based on the CLSI (11–13) and by considering the availability and frequent prescriptions of these drugs for the treatment of urinary tract infections in the study area.

**Extended spectrum β-lactamase detection:** Initial screening for ESBL was done by the diameters of zones of inhibition produced by either Ceftazidime (30 µg) or Cefotaxime (30µg) from the AST on MHA according to the CLSI screening criteria. These breakpoints indicated of suspicion for ESBL production were: for Ceftazidime (30 µg) ≤ 22mm and for Cefotaxime ≤ 27mm. After this initial screening was done, phenotypic detection of ESBL production was confirmed by Combined Disk (Double Disk Potentiate) test according to CLSI (13).

**Quality control:** Specimen collection was made following the recommended approach. We strictly followed the manufacturers' instruction and bacteriological standard procedures during culture media preparation and AST testing. The standard reference bacteria strains such as *E.coli* (ATCC 25922), *P. aeruginosa* (ATCC 27853) and *S. aureus* (ATCC 25923) were used for quality control of culture and antimicrobial susceptibility tests.

**Data analysis:** Data were entered, cleaned and analyzed by using Statistical Software for Social Package (SPSS) version 23. Generated data were compiled by frequency tables and figures and other statistical summary measures. The proportion of uropathogenes was calculated. Similarly, the proportion of AMR to a specific drug was calculated. Chi-square (*X^2^*) was considered to find out factors associated with culture positive urine samples and statistical significance was set at *p* value < 0.05.

**Ethical approval**: An ethical clearance letter was obtained from the institutional review board (IRB) of college of medicine and health science, Bahir Dar University. Following wellversed about the purpose and importance of the study, informed written consent was obtained from all the study participants. Information obtained during this study was kept confidential and used only for the study purpose. Bacteriological positive results were communicated for health professionals attending the pregnant women for better management.

## Results

**Demographic and clinical characteristics of the study participants:** Of the total 384 study participants, information from eight of the participants, including the collected urine volume was considered insufficient and excluded from the final analysis. Therefore, data of 376 pregnant women were included in this study. The majority of the study participants were in the age group of 26–35 years 183(48.7%), married 368(97.9%), don't write or read 135(35.9%), urban dwellers 344(91.5%) and multiparous 239(63.6%). Approximately 88(23.4%), 147(39.1%) and 161(42.8%) of the study participants were in their third trimester, had previous history of UTI and their income was between 200–400 USD, respectively (Table 1 and 2).

**Table 1:** Socio-demographic Characteristics of Study Participants in Edna Adan Hospital, May, 2019

Variable	Number(%)
*Age in years*	
15–25	175 (46.5)
26–35	183(48.7)
36–45	18(4.8)
*Residence*	
Urban	344(91.5)
Rural	32(8.5)
*Educational status*	
Unable to read and write	135(35.9)
Only read and write	52(13.8)
Primary School Completed	44(11.7)
Secondary School Completed	62(16.5)
University or College Completed	83(22.1)
*Monthly income*	
Less Than 200 USD	161(42.8)
200–400 USD	82 (21.8)
400–600 USD	101(26.9)
600–800 USD	12(3.2)
Greater Than 800 USD	20(5.3)
*Gravidity*	
Multigravida	287(76.3)
Primigravida	89(23.7)
*Parity*	
Nulipara	70 (18.6)
One	67()17.8
Multipara	239(63.6)
*Gestation period*	
First Trimester	88(23.4)
Second trimester	127(33.8)

Third trimester	161(42.8)

**Table 2:** Clinical characteristic of the study participants at Edna Adan Hospital, May, 2019

Characteristics	Yes, n (%)	No, n (%)
Dysuria	148(39.4)	228(60.6)
Increased Frequency	92(24.5)	284(75.5)
Urgency	93(24.7)	283(75.3)
Hemauria	19(5.1)	357(94.9)
Fever & Chills	89(23.7)	287(76.3)
Flank Pain	80(21.3)	296(78.7)
History of Catherization	40(10.6)	336(89.4)
History of UTI	147(39.1)	229(60.9)
History of Diabetes Mellitus	28(7.4)	348(92.6)
History of Hospitalization	25(6.6)	35 (93.4)
History of Antibiotic use	86(22.9)	290(77.1)

**Figure 1:**
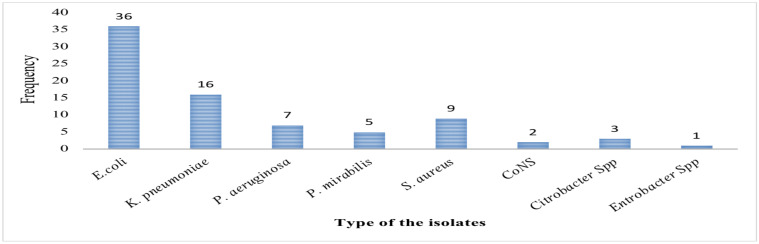
Frequency and type distribution of the identified uropathogens among pregnant women at EAH, 2019.

The identified uropathogens: Of the total 376 processed urine samples, 79 (21%) were noted with a colony count of ≥10^5^ cfu/ml of urine, what is known as significant bacteriuria. Majority at 58(73.4%) were Gram-negative while the remaining, 21(26.6%) of the isolate were Gram-positive. The most predominant isolate was *E.coli*, 36(45.6%) followed by *K.pneumonea* 16 (20.3%) and *S. aureus* 9 (11.4 %) (Figure 1). The distribution of isolates among symptomatic and asymptomatic pregnant women for UTI was at 55(69.6), and 24(30.4), respectively.

**Antimicrobial resistance and ESBL profile of the isolates:** With regard to the AMR profile of the isolates, majority of Gram-negatives showed resistance against ampicillin at (87%), amoxicillin (85%), cefotaxime (57%) and cephalexin at (52%) and nitrofurantion was found to be effective against most of the isolates. All *S. aureus* isolates were found 100% sensitive to amoxicillin-clavulanic acid, ceftriaxone, nitrofurantion, ceftazidime, norfloxacin, cefotaxime and cephalexin (Table 3 and Table 4).

**Table 3:** Proportion of ESBL production among the isolates (n=79), May 2019

Type of the Isolates	Number	%	ESBL producers n (%)	Non-ESBL producers n (%)
*E.coli*	36	45.5	14(38.8)	22(61.2)
*K.pneumonae*	16	20.3	5(31.3)	11(68.5)
*P.aeruginosa*	7	8.9	3(42.9)	4(57.1)
*P.mirabilis*	5	6.3	1(20)	4(80)
*S.aerues*	9	11.4	-	9 (100)
*CoNs Spp*	2	2.5	-	2 (100)
*Citrobacter Spp*	3	3.8	1(33.3)	2(66.7)
*Entrobacter Spp*	1	1.3	1(100)	0

Total	79	100.0	25 (31.6)	54 (68.4)

**Table 4:** Antimicrobial Resistance profile of the bacteria uropathogens among pregnant women at Edna Adan Hospital, May 2019

Antibiotics Used	Bacterial Isolates							

	*E.coli (n=36)*	K. pneumoniae *(n=16)*	*P.aeruginosa* *(n=7)*	S. aureus *(n=9)*	*Proteus* *Spp* *(n=5)*	*Citrobacter* *Spp* *(n=3)*	CoNS (n=2)	*Enrobacter* *Spp* *(n=1)*
Ampicillin	18(50 %)	14(87.7 %)	5(72 %)	7(33 %)	2(40 %)	2(34.7%)	1(50 %)	0(0 %)
Amoxicillin	21(59 %)	7(44 %)	6(86 %)	4(45.4 %)	2(60 %)	0(0.00 %)	0(0.00%)	0(0.00 %)
Amxclavulanic acid	32(11.1%)	0(0.00%)	3(57.1 %)	0(0.00 %)	0(0.00 %)	0(0.00%)	0(0.00%)	0(0.00 %)
Nitrofurantoin	0(0.00 %)	0(0.00 %)	0(0.00 %)	0(0.00 %)	0(0.00 %)	0(0.00%)	1(50 %)	0(0.00 %)
Ceftazidime	5(13.9%)	6(37.5%)	4(57.1%)	1(11.1 %)	0(0.00 %)	0(0.00%)	0(0.0 %)	0(0.00 %)
Norfloxacin	28(22.3 %)	5(31.2%)	3(57.1 %)	0(0.00 %)	3(40 %)	0(0.00 %)	0(0.0 %)	0(0.00 %)
Cefotaxime	22(61.1.8%)	9(43.6 %)	4(57.1 %)	0(0.00 %)	0(0.00%)	0(0.00 %)	1(50 %)	0(0.00 %)
Cephalexin	17(47.2 %)	9(43.6%)	3(42.9 %)	0(0.00 %)	1(80 %)	1(33.3%)	0(0.00%)	0(0.00 %)

Ceftriaxone	30(83.3 %)	14(87.5 %)	0(0.00 %)	9(100 %)	4(80 %)	3(100 %)	1(50 %)	0(0.00 %)

The overall proportion of ESBL production was at 25 (32%). Specifically, *P. aeruginosa* 3(42.9%), *E.coli* 14*(38.8%) K. pneumonae*, 5(31.3%), *P. mirabilis*, 1(20%), *Citrobacter Spp* 1(33.3%) and *Entrobacter Spp* at 1(100%) were found positive for ESBL production. However, among *S. aureus* and *CoNS* isolates there was no ESBL production reported.

Factors associated with significant Bacteriuria: Previous history of UTI, monthly income, educational status and having dysuria were found to be significantly associated with culture positive urine among pregnant women in Edna Adan Hospital (*p<0.05*). The rest variables didn't show statistical association.

## Discussion

Bacterial UTI is one of the common causes for seeking clinical service among pregnant women in different settings. Effective management of patients suffering from bacterial UTIs commonly relays on the identification of the type of the uropathogen that caused the disease and the selection of an appropriate antimicrobial agent effective to the organism in question (14, 15, 16).

In the present study a total of 376 pregnant women were included for urine bacteriological culture and AST analysis. The proportion of significant bacteriuria was at 79(21%). Although there was no previous study in our setting to compare the finding, the result was found relatively higher than reports in Ethiopia (8.5%) (6), Sudan (12.1%)(8) and Saudi Arabia (12%) (9). In contrast, our result was also lower than the prevalence reported in Ghana (29.9%) and Nepal (31.4%)(17, 18), respectively. The variation of the reported prevalence of significant bacteriuria among pregnant women across different studies from one country to other or among region of the same country might be attributed to the difference sample size, geographical variations, host factors and social habit of the community and health education practice, environmental conditions and the standard of personal hygiene.

In the present study, Gram-negatives were more prevalent at 58(73.4%) than Gram-positive isolates. This finding is comparable to other studies done in Dire Dawa, Ethiopia where (73.1%) of the isolates were reported to be Gram-negative (10). Further, our finding is in-line with the fact that Gram-negative bacteria are the most predominant uropathogens that usually sourced from the bowel and ascend to the urinary tract. They have also unique structures (like, pilus adhesions) which help the bacteria for attachment to the uroepithelium lining and prevent them from urinary lavage, allowing for multiplication and tissue invasion resulting in invasive infections during pregnancy (1–3). *E.coli*, 36(45.6%) was the most predominant identified uropathogen followed by *K.pneumonea* at 16 (20.3%). It is well documented knowledge that most UTIs are caused by Gram-negative bacteria like *E. coli* and *Klebsiella spp.* Specifically, *E. coli* is certainly the most common bacteria isolate from urine samples in both outpatients and inpatients of both sexes, and this finding is in agreement with others studies too (5–10). Moreover, in the present study among Gram-positive uropathogens *S. aureus* was found predominant bacteria which accounted for about 9(11.4%). This finding is consistent with the studies conducted in Gondar University teaching hospital, Ethiopia (5).

With regard to the antimicrobial sensitivity profile of the isolates, (87.7 %) and (100%) were found resistant to Ampicillin and Amoxicillin, respectively. Majority of Gram-negative isolates found resistant to ampicillin at (87%), Amoxicillin (85%), Cefotaxime (57%) and Cephalexin at (52%). This level of AMR might be attributed by a number of issues including over and misuse of antimicrobials in the study area where there is weak regulatory practice and inadequate bacteriological surveillance due to lack of routine antimicrobial susceptibility testing facilities. Most of the antimicrobials listed are freely available in local market and people could purchase and use them without prescription. This would also play its part for high-level antimicrobial resistance reported in this study. In the present study, almost all isolates of *S.* aureus *and Citrobacter Spp* were found sensitive for the drugs they were tested. Similarly, Amx-Clavulanic acid and nitrofurantoin were found to be comparatively effective against the uropathogens. Low-level of resistance, specifically against nitrofurantoin might be due to its narrow range of clinical indications, which results in less usage. Nitrofurantion was found to be the most effective drug against *E.coli* followed by Amx-Clavulanic acid. Hence, Nitrofurantoin can be considered in the present study as one of the drugs of choice to treat UTI in pregnant women where multi-drug resistant uropathogens are prevalent. Our finding is in agreement with a report by (19) that stated nitrofurantoin to be the most effective drug against *E. coli.*

ESBL-producing *uropathogenes* have become a problem worldwide. Dissemination of ESBLs compromises the activity of broad-spectrum antibiotics creating major therapeutic difficulties with a significant impact on the outcomes for patients The magnitude of ESBL producing organism among clinical isolates vary greatly worldwide and changing over time (19–21). The present study revealed that, the overall prevalence of ESBL production was at 25 (32%). A similar study by (21) in India reported 48.3% level of ESBL proportion which is higher than our report. In the present study specifically, *P.aeraginosa* was found to be the higher ESBL proportion at 42%, followed by *E.coli* at 38.8%. This finding was relatively lower than a study conducted in Bangladesh that reported ESBL profile of *E. coli* at (60%) and *K. pneumonea* at (40%) (22). However, Behrooozi et al. study in Iran showed higher reports for these isolates at (21%) and (12%), respectively (23).

Our study has some confines that should be considered while interpreting the data. In this study authors did not attempt to identify non-bacterial uropathogens and no serotyping was also done for some isolates due to resource limitation. In addition, the molecular characteristics of the isolates were not performed. Further, urine sample was collected once regardless of the participants' period of gestation, this could potentially influence to reveal the actual status of SBU during the entire period of pregnancy.

In conclusion, this study revealed a prevalence of 21% significant bacteriuria among pregnant women in EAH. *E. coli* was the most predominant isolate followed by *Klebsiella Spp* and *S. aureus*. A large number of the isolates were found resistant to the commonly prescribed antimicrobials. However, low level of resistances was documented against Norfloxacin, Nitrofurantion and Amoxicillinclavulanic acid and hence could be considered as an empirical therapy for UTI in the study area. The overall proportion of ESBL production was at 31.6%; specifically 38.8%, 31.3% and 42.6.5% of E.coli, *K. pneumoniae* and *P. aeruginosa* were found positive for ESBL production. Low level of income, previous history of UTI and having dysuria were found significantly associated with SBU.

Therefore, a rational use of antimicrobials, collaborative regular surveillance of pathogens associated with UTI among the pregnant women should be considered to better manage the case among this segment of the population in the study area. Empirical antibiotic selection should be geared based on the knowledge of the local prevalence of uropathegenes and their respective AST patterns. Further study should be considered to molecularly characterize the isolates and to identify bacteriological factors associated with ESBL production.
